# The Venereal Disease of the Horse

**Published:** 1841-10

**Authors:** 


### Art. XIV.

*Die Venerische Krankheit der Pferde.* Von J. L. Harthausen, der Arznei-und Wund-Arzneikunde Doctor, &c. &c.—*Breslau,* 1839.

*The Venereal Disease of the Horse.* By Dr. J. L. Harthausen, &c. —*Breslau,* 1839. 8vo, pp. 60.

This is a notice of a disease occurring among stallions and brood-mares, which it is stated " is developed under peculiar miasmatic and individual relations, and is then communicated, only however by contagion, in the act of copulation." It is characterized " by marked and continued depression of the nervous power; by mucous discharge, especially from the parts of generation; by disturbed functions of the cutaneous, lymphatic, and glandular systems; by consequent formation of ulcers upon and of knots in and under the skin; by paralysis of the hinder extremities; and finally, by fatal emaciation." This affection, although probably of prior occurrence, does not appear to have been distinctly recognized before the year 1818; but between this period and the year 1821 a disease with symptoms as above mentioned seems to have prevailed in Styria. A similar affection showed itself in Bohemia in the year 1826-7, and continued for several years, and both before and since the same disease appears to have visited West Prussia, Lithuania, Hanover, and part of Switzerland. In the spring of the year 1833 two sporadic and isolated cases came to the knowledge of the official authorities in the village of Steindorf, on the borders of Austrian Silesia. Three years subsequent to this period, during an epidemic of catarrhal rheumatism, the strangles (glandular disease of the throat) being also prevalent, the disease occurred, pretty generally spread and very severe, in the department of Neisser, always however only after a previous covering, and in far greater proportion after covering by private stallions than by those belonging to studs. It is remarkable that, notwithstanding careful examination, not the slightest trace of disease was detected in the greater number of the stallions. The disease again appeared during the month of March of the year 1838, but was checked by the observance of appropriate regulations. (pp. 8-11.)

The morbid appearances in the genital organs usually show themselves in a few weeks, sometimes however in a few days, after the act of copulation; but a peculiar depression, resembling narcotism, may be observed within the first three days. This state of the nervous system has been confounded, it would seem, with that peculiar depression observed to follow the act of coition, which has given rise to the aphorism, *post coitum animal triste;* and the symptoms of local irritation, the frequent micturition and looseness, the repeated switching of the tail, the restless state, and alternate raising and extension of the hind-foot, have been no less confounded with the results of the natural excitement of the genitals at these times. In the more severe cases, after a certain time, there is swelling of the parts of generation, without either pain or sensibility on pressure, heat or increase of temperature, but attended rather with a sense of coldness to the touch. This swelling commences at the under part of the pouch or entrance of the vagina, from which it spreads round the orifice, and not unfrequently to the perineum and udder, &c. Upon closer examination of the parts, the mucous membrane of the vagina is found to be relaxed, slightly reddened, moderately swollen, and covered with a viscid mucus resembling the white of egg.

"The redness shows itself, without increase of the natural temperature, as an equally diffused tinge, which, here and there, at indeterminate points, higher and deeper, is traversed in an isolated course by injected capillary vessels. These are collected in groups, and form, though less frequently, pale wine-red spots from a quarter to half an inch in diameter. When they occur in the *fossa navicularis* of a deeper red and wreathed, a small black point, the orifice of a sebaceous crypt, may be observed in the centre, from which coagulated particles of caseous matter may be pressed out in unusual quantity." (p. 12.)

The discharge from the vagina, which commonly makes its appearance before the swelling, is at first neither very abundant nor discoloured, and is apparently nothing more than an increased secretion of the natural mucus; but in the further progress of the disease it becomes not only viscid, ropy, and of a reddish-yellow colour, but the secretion is gradually altered in its characters. From being bland and affording no trace of either acid or alkaline properties, it is found to contain free phosphoric acid, showing great tendency to coagulate and form incrustations, so that the cleft of the vagina and the neighbouring parts frequently become covered with yellow or red-brown amber-coloured crusts. The discharge of the mucus, which had been frequently so considerable as thoroughly to moisten the tail and hind-feet, becomes now periodical, a viscid secretion collecting within the vagina, and only exuding therefrom on the movement of the animal. The vaginal membrane becomes flaccid and wrinkled, and of a pale yellow, grayish-white, and sometimes of a livid appearance. The cold œdematous swelling of the labia (*Tasche*) and perineum sinks remarkably, forming loose folds, and the relaxation extending so far that in the greater number of cases the cleft of the vagina gapes and the clitoris becomes exposed. In the natural course of the disease, or in consequence of the absorption of the vitiated secretion, favoured by its retention within the vagina, there appear, sparingly scattered over the external surface of the labia, small phlyctænæ, which rapidly pass into chapped ulcerations incrusted with lymph. The crusts are quite superficial, circular, and never exceed the size of a fourpenny piece, very rarely becoming confluent or running into each other, but remaining distinct like the crusts of the varioloid eruptions. Similar ulcerations have been elsewhere observed situated on the mucous membrane of the vagina; but Dr. Harthausen has never seen an example of this in his own neighbourhood, although he has noticed that the mucous membrane at this period of the disorder has assumed a marbled appearance from white and livid spots on its surface. The extension of these ulcerations to other parts follows a peculiar course, the eruption spreading from the genital organs forward to the rest of the body, and occupying the head and throat in countless numbers, but never appearing on the extremities. After the healing of the ulcers, innumerable isolated milk-white spots deprived of the hair are left behind, so that the horse appears as if speckled. These spots never present any inequality of surface, pitting, or marks of cicatrization, the naked skin being on the contrary perfectly smooth, and differing from the healthy skin in nothing but its conspicuously milk-white colour. (pp. 13-4.)

Accompanying and frequently preceding the local symptoms is a general failure or depression of the nervous power; the senses become blunted, the expression of the animal is dull and heavy, motion of any kind is effected with difficulty, and there is evident loss of power in the loins and hinder extremities. This state cannot be attributed to debility arising from the profuseness of the discharge or from emaciation, but seems to be owing, as Dr. Harthausen thinks, purely to an impression made on the nervous system, since frequently when most severe the discharge has been by no means extensive. Neither does it appear to be owing to absorption of the altered secretion, as it occasionally precedes the occurrence of the local symptoms.

After the ulceration has continued for some time, knots or tubercles of various dimensions are developed beneath the skin: some of these are as large as a dollar in circumference, but the greater number do not exceed the transverse diameter of a walnut. They are smooth, firm, and elastic, circular in form, and sharp-edged, and altogether similar to those produced by the bites or stings of insects. There is neither pain on pressure, nor any remarkable redness or heat of the parts, and neither fever nor any appreciable premonitory symptom has been observed to precede their occurrence. They first make their appearance upon the haunches, then upon the head and throat, afterwards upon the shoulders, breast, and other parts of the body, but have never yet been observed on the extremities. When they open on the surface, which very rarely occurs, a sero-lymphatic matter exudes; after a time this discharge dries up and the tubercles disappear. The duration of these, as well as of the tubercles from which no discharge takes place, is uncertain, and the latter frequently disappear from the place which they occupy as suddenly as they had been previously developed there. (pp. 16-7.)

In proportion to the duration and number of the tubercles beneath the skin, and the ulcers on the surface, is the liability of the large glands to take part in this disease: swelling of these glands takes place, and in very many instances a chronic glandular affection is the consequence, which not unfrequently terminates in glanders, and, when the formation of the tubercles has prevailed throughout the course of the disease and the morbid secretion has been checked, in farcy, rapidly followed by a fatal result. Glanders and farcy are however by no means the most usual terminations of the disease. The ulcers and tubercles having continued for a few weeks, and gradually gaining firmer hold upon the system, a general state of relaxation more commonly ensues. Twitches or shiverings (zuckende Bewegungen, wie bei den Empfindungen des Frostes), never of the whole body, but occupying individual layers of muscles only, and in single muscles resembling the catchings produced by galvanism, may be observed. These partial rigors are accompanied by extreme weakness of the posterior parts, and are followed by paralysis, varying in the site which it occupies and the degree to which it attains without any regularity or apparent dependence upon lesion of the central organs. In some singular instances the loss of power affects one ear or the under lip, in others the extreme portion only of these organs, so that the tip of the ear, for instance, is bent at an acute angle as if the cartilage was broken: from this circumstance Dr. Harthausen infers that the paralysis does not exclusively proceed from the nervous centres, but may be owing also to affection of the extremities of the nervous trunks. Finally, the paralytic affection varies as much in the form which it assumes as in degree, in situation, and in the symptoms accompanying it. Cases occur in which its origin seems to be respectively in the brain, in the spinal marrow; in the nervous centres, in their peripheral extremities; while in some instances sensation and motion are equally impeded, in others the motor nerves alone are affected. (p. 19.) In a few weeks the animal sinks under these symptoms; the ulcers and tubercles in some instances declining before death by desquamation of the skin, in others gradually disappearing. The entire duration of the affection is from four to nine months, during the whole of which time the pulse continues slow and sluggish, almost without change, to the end.

The foregoing account refers to the disease as it appears in the mare; as it appears amongst stallions it differs in that the constitutional symptoms precede the local affection of the genitals,—emaciation, weakness of the loins and hinder extremities, paralysis of different parts, and symptoms of a diseased state of the lymphatic and glandular systems, preceding the occurrence of swelling of the testicles, vesicular eruption in the penis, &c. This is however stated to be the case only where the disease is of spontaneous origin, or arises without evident cause; where it has been communicated by contact the progress is, as in the mare, from without inwards. The first symptoms of the infection are swelling of the prepuce and the appearance of small miliary vesicles on the penis, which give rise to slight excoriations and the formation of thin lymphy incrustations. The vesicles are usually first formed at the extreme point of the urethra, and, as well as the excoriations, are of the same pale rose-red colour as the lining membrane of the canal; they are also visible even from the commencement, in greater or less number, in the fold of the prepuce, at the base of the glans penis. Subsequently there is swelling of the testes, followed by irritation of the lymphatics of the groins and the development of superficial lymphatic tubercles upon the skin about the loins, and the disease then pursues its course with the formation of superficial ulcerations, &c., as before described in the mare.

The preceding details are sufficient to characterize this severe and dangerous affection should it be found to exist in this country; but further and closer research is required before the question as to the origin of the symptoms from a specific virus can be determined. It would seem to be frequently of spontaneous origin, both in the horse and in the mare, and at the same time appears to be communicable from the one to the other. There is however some cause to think that this communication may take place rather as a consequence of the local excitement depending upon the generative act, under a peculiar epidemic constitution predisposing to the development of glandular disease, than as a result of the absorption of a specific poison. The appearances after death, as may be conjectured, present nothing peculiar, and throw little light on the nature of the disease. Those most frequently observed are morbid alterations of the mucous membranes of the absorbent vessels and glands, and of the brain and spinal marrow. The changes of structure in the brain, and especially in the spinal marrow, are the most important, there being few cases recorded in which softening of the substance of some portion of these organs, congestion of the membranes, or serous effusion and infiltration did not occur.

---

